# Use of Two-Part Regression Calibration Model to Correct for Measurement Error in Episodically Consumed Foods in a Single-Replicate Study Design: EPIC Case Study

**DOI:** 10.1371/journal.pone.0113160

**Published:** 2014-11-17

**Authors:** George O. Agogo, Hilko van der Voet, Pieter van’t Veer, Pietro Ferrari, Max Leenders, David C. Muller, Emilio Sánchez-Cantalejo, Christina Bamia, Tonje Braaten, Sven Knüppel, Ingegerd Johansson, Fred A. van Eeuwijk, Hendriek Boshuizen

**Affiliations:** 1 National Institute for Public Health and the Environment, Bilthoven, The Netherlands; 2 Biometris, Wageningen University and Research Center, Wageningen, The Netherlands; 3 Department of Human Nutrition, Wageningen University and Research Center, Wageningen, The Netherlands; 4 Nutritional Epidemiology Group, International Agency for Research on Cancer, Lyon, France; 5 Department of Gastroenterology and Hepatology, University Medical Center Utrecht, Utrecht, The Netherlands; 6 Genetic Epidemiology Group, International Agency for Research on Cancer, 150 cours Albert Thomas, Lyon, 69008, France; 7 Andalusian School of Public Health, Granada, Spain; 8 CIBER de Epidemiología y Salud Pública (CIBERESP), Barcelona, Spain; 9 WHO Collaborating Center for Food and Nutrition Policies, Department of Hygiene, Epidemiology and Medical Statistics, University of Athens Medical School, Athens, Greece; 10 Department of Community Medicine, University of Tromsø, N-9037, Tromsø, Norway; 11 Department of Epidemiology, German Institute of Human Nutrition Potsdam-Rehbrücke, Potsdam, Germany; 12 Department of Odontology, Umeå University, Umeå, Sweden; Tianjin University, China

## Abstract

In epidemiologic studies, measurement error in dietary variables often attenuates association between dietary intake and disease occurrence. To adjust for the attenuation caused by error in dietary intake, regression calibration is commonly used. To apply regression calibration, unbiased reference measurements are required. Short-term reference measurements for foods that are not consumed daily contain excess zeroes that pose challenges in the calibration model. We adapted two-part regression calibration model, initially developed for multiple replicates of reference measurements per individual to a single-replicate setting. We showed how to handle excess zero reference measurements by two-step modeling approach, how to explore heteroscedasticity in the consumed amount with variance-mean graph, how to explore nonlinearity with the generalized additive modeling (GAM) and the empirical logit approaches, and how to select covariates in the calibration model. The performance of two-part calibration model was compared with the one-part counterpart. We used vegetable intake and mortality data from European Prospective Investigation on Cancer and Nutrition (EPIC) study. In the EPIC, reference measurements were taken with 24-hour recalls. For each of the three vegetable subgroups assessed separately, correcting for error with an appropriately specified two-part calibration model resulted in about three fold increase in the strength of association with all-cause mortality, as measured by the log hazard ratio. Further found is that the standard way of including covariates in the calibration model can lead to over fitting the two-part calibration model. Moreover, the extent of adjusting for error is influenced by the number and forms of covariates in the calibration model. For episodically consumed foods, we advise researchers to pay special attention to response distribution, nonlinearity, and covariate inclusion in specifying the calibration model.

## Introduction

Dietary variables are often measured with error in nutritional epidemiology. In such studies, usual dietary intake is assessed with instruments such as, food frequency questionnaire and dietary questionnaire [1]–[3]. In these instruments, the queried period of intake ranges from several months to a year. As a result, these instruments are prone to error caused by difficulties to recall past intake of foods or food groups, the frequency of consumption, and the portion size. In general, the measurement error in usual dietary intake can either be systematic or random. Systematic error occurs when an individual systematically overestimates or underestimates dietary intake, whereas random error is due to random within-individual variation in reporting of dietary intake [1], [4]. The random error attenuates the association between dietary intake and disease occurrence, whereas systematic error can either attenuate or inflate the association.

As a case study, we used the European Prospective Investigation on Cancer and Nutrition (EPIC) study. In EPIC, country-specific dietary questionnaires, hereafter DQ, were used to measure usual intake of various dietary variables or groups of dietary variables in different participating cohorts. With DQ measurements for usual intake, an association parameter estimate that relates usual intake to disease occurrence is often biased, mainly towards the null [4]–[6].

Regression calibration is the commonly used method to adjust for the bias in the association between usual intake and disease occurrence, due to measurement error in the DQ. Regression calibration involves finding the best prediction of true usual intake given DQ measurements and other error-free variables [7]. The prediction is further used as a proxy for true usual intake in the disease model that relates dietary intake to disease occurrence. Regression calibration requires a calibration sub-study, where unbiased measurements are taken. Some prospective studies therefore include a calibration sub-study that can either be internal or external. Internal calibration study consists of a random sample from the main study population, as was the case in the EPIC, whereas external calibration sub-study consists of subject not in the main-study but with similar design as the main-study [8]. In the calibration sub-study, unbiased reference measurements are collected by short-term reference instruments, such as food records or 24-hour dietary recalls. The reference measurements can be used as the response in the calibration model to predict true usual intake. In the EPIC study, regression calibration can also adjust for systematic error in DQ measurements due to the multicenter component of the EPIC study, as described in [9], [10]. In the EPIC calibration sub-study, a 24-hour dietary recall, hereafter 24-HDR was used as the reference instrument. For each subject in the calibration sub-study in the EPIC, only one reference measurement was available [11]. For foods that are not consumed daily, 24-HDR measurements would contain many zeroes for many individuals. Handling these zeroes poses a challenge in the calibration model [12]–[15]. The excess zeroes can be handled with regression calibration in a two-step approach, where the consumption probability and the consumed amount on consumption days are modeled separately [13]. We refer to this model as two-part regression calibration.

The currently published studies on two-part regression calibration method require epidemiologic studies with at least two replicate reference measurements per subject [13]–[15]. Given the design of the EPIC study with a single measurement per individual, however, these calibration models cannot be applied directly. Moreover, the performance of the calibration models in a study design such as EPIC for episodically consumed foods has not been studied exhaustively. Further, the effect of variable selection on the performance of a two-part calibration model has not yet been studied fully. The standard theory of selecting covariates into the calibration model states that confounding variables in the disease model must be included in the calibration model together with the covariates that only predict dietary intake but not the risk of the disease [14], [16].

To fill the aforementioned gaps, we developed a two-part regression calibration model to adjust for the bias in the diet-disease association, due to measurement error in self-reported episodically consumed foods, when each subject in the calibration sub-study has only a single reference measurement. The second goal was to assess the effect of reducing the number of variables in the two-part calibration model with the covariates selected based on the standard theory. As a working example, we studied the association between intakes of each of the three vegetable subgroups: leafy vegetables, fruiting vegetables, and root vegetables, on all-cause mortality as reported in the EPIC. We described how to handle the excess zeroes, the highly skewed-heteroscedastic non-zero reference measurements, non-linear relations in the calibration model, and how to select covariates into the calibration model. We showed that a suitably specified two-part calibration model adjusts for the bias in the diet-disease association caused by measurement error in self-reported intake in EPIC study. We further showed that the extent of adjusting for the bias is much influenced by how the calibration model is specified.

## Materials and Methods

### Ethics Statement

All participants who agreed to join the EPIC study signed an informed written consent. The study was approved by the Institutional Review Board of the International Agency for Research on Cancer and local institutional review boards of each participating center.

### Study subjects

EPIC is an on-going multicenter prospective cohort study to investigate the relation between diet and the risk of cancer and other chronic diseases. The study consisted of 519,978 eligible men and women aged between 35 and 70 years and recruited in 23 centers in 10 Western European countries [11], [17]. The 10 participating countries were: France, Italy, Spain, United Kingdom, Germany, The Netherlands, Greece, Sweden, Denmark, and Norway. The study populations comprised of heterogeneous groups. In most centers, study populations were based on general population while some consisted of participants in breast screening programs (Utrecht, The Netherlands; and Florence, Italy), teachers and school workers (France) or blood donors (certain Italian and Spanish centers). In Oxford, most of the cohort was recruited among subjects with interest in health or on vegetarian eating. Only women were recruited in France, Norway, Utrecht (The Netherlands) and Naples [18]. Information on usual dietary intake, lifestyle, environmental factors and anthropometry was collected from each individual at baseline. The dietary intake information was assessed with different dietary history questionnaires, food frequency questionnaires or a modified dietary history developed and validated separately in each participating country [17]. The questions asked in the questionnaires included the frequency of consumption over the past 12 months preceding the administration, categorized into the number of times per day, per week, per month or per year. A calibration sub-study was carried out within the entire EPIC cohort by taking a stratified random sample of 36,900 subjects. In the calibration sub-study, a 24-HDR was administered once per subject using a specifically developed software program (EPIC-SOFT) designed to harmonize the dietary measurements across study populations [19].

We used EPIC dietary intake data for leafy vegetables, fruiting vegetables and root vegetable sub-groups as a working example. We further assumed measurements on the 24-HDR (in g/day) as the unbiased reference measurements and those on the DQ as the biased main-study measurements. We excluded subjects with missing questionnaire data, missing dates of diagnosis or follow up, in the top and bottom 1% of the distribution of the ratio of reported total energy intake to energy requirement. We further excluded subjects with a history of cancer, myocardial infarction, stroke, angina, diabetes or a combination of these diseases at baseline. As a result, data for 430,215 subjects were eligible for the analyses. In the analysis, the data from the following centers were excluded: Umeå and Norway for leafy vegetables and Norway for fruiting vegetables. The decision to exclude these data was based on the inclusion criteria as stipulated in the EPIC analysis protocol.

### Disease model

In epidemiological studies, the interest is mainly in the association between an exposure and disease occurrence. In our working example, we were interested in the association between intake of vegetable subgroups and all-cause mortality. If the true usual intake of vegetable subgroups were known, then a generalized linear disease model would be:
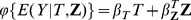
(1)where *Y* is a disease outcome, here, an indicator for mortality, *T* is true usual dietary intake of a vegetable subgroup, **Z** is a vector of error-free confounding variables and *φ* is a function linking the conditional mean and the linear predictor. The coefficient 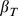 quantifies the association of interest and 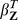 is a vector of coefficients for the confounding variables. If dietary intake is measured with error, then 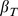 would mostly be underestimated. Therefore, a researcher should adjust for the bias in estimating 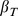 due to measurement error in DQ.

### Regression calibration model

Regression calibration is the most commonly used method to adjust for the bias in estimating 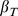 (i.e., diet-disease association) due to measurement error in the DQ. To describe regression calibration, we denote reference measurement from 24-HDR by *R*, main-study measurement from DQ by *Q*, and the covariates that only predict vegetable intake and not mortality by ***C***. Then, a set of all covariates that possibly relate to usual intake is given by 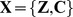. Regression calibration involves finding the best prediction of true usual intake given DQ measurement and other covariates [14]. The mean predictor from regression calibration is denoted by 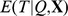. A major challenge in fitting the calibration model is that true usual intake is not only unobservable but also cannot be measured exactly. To circumvent this, a reference measurement is required in place of the latent true intake. The reference measurement should be unbiased for true intake, and should be measured with errors that are uncorrelated with the errors in the DQ measurements. We, therefore, made two strong assumptions. First, we assumed the short-term measurement from the 24-HDR to be an unbiased measurement for true usual intake. Second, we assumed the errors in the 24-HDR measurements to be uncorrelated with the errors in the DQ measurements. We denote the calibration model by:
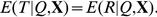
(2)


We assumed in model (2) that measurement error in *Q* does not provide extra information about *Y* other than that provided by *T*. The measurement error in *Q* is, therefore, said to be non-differential. In model (2), *R* is modeled as a function of *Q* and ***X*** using standard regression methods, where a suitable distribution for the error terms and a suitable parametric form of each covariate in ***X*** is chosen.

In this work, we considered only the case of a single dietary intake variable measured with error. In our data, the correlation between the vegetable subgroups and the other confounders, as measured by the questionnaire, were low justifying their omission, as the contamination effect of the measurement error in these variables on the correction factor for our dietary intake of interest would be negligible.

### Excess zeroes, heteroscedasticity and skewness in reference measurements

Vegetable subgroups considered in this study are not consumed daily. This results in many zero reference measurements reported on the 24-HDR. As a result, the reference measurements have a mixture of zeroes for non-consumers and positive intake for consumers. The excess zeroes pose challenge in regression calibration, with the reference measurements as the response. To handle these excess zeroes, we used a two-part approach to build a regression calibration model. In the first part, the consumption probability as reported in the 24-HDR is modelled. In the second part, the consumed amount on consumption occasion is modelled [13]. The first part involves discrete data and can be modeled either with logistic or probit regression, where the probability of consumption depends on a given set of covariates. In the second part, plausible family of densities for the consumed amount on consumption occasion can be assumed [20]. The GLM model for the consumption probability is parameterized as.


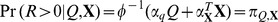
. Similarly, the GLM model for the consumed amount is parameterized as 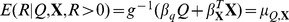, where 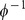 can be either inverse-logit or inverse-probit function and 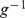 can be an inverse of any plausible link function. Thus, the calibration model (2), adapted to two-part form to handle the excess zeroes in the response is parameterized as 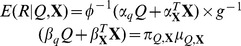. The true usual intake can thus be predicted from this model. We denote the prediction from this two-part calibration model by
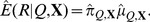
(3)


Another challenge is how to handle distribution for the consumed amount that is commonly right-skewed with heteroscedastic variance. To handle heteroscedasticity, we applied a generalized linear modeling (GLM) approach in a regression calibration context. In the GLM approach, the variance is linked to the mean as 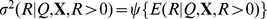, where 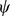 is a function that links the conditional variance with the conditional mean of reference measurement in the model for consumed amount, 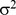 denotes the conditional variance, and 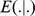 denotes the conditional expectation [21]. The advantage of GLM approach is that the consumed amount can be predicted directly without transforming the data. To determine the optimal relation between the conditional variance and the conditional mean, the GLM model is parameterized using a class of power-proportional variance functions as follows: 
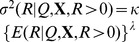
, where *κ* denotes the coefficient of variation, *λ* is a finite non-negative constant. This power variance function can be rewritten in a linear log-form as follows:
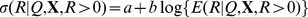
(4)where 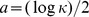 and 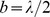. In model (4), *λ* equals zero refers to a classical nonlinear regression with constant error variance, *λ* equals one refers to a Poisson regression with the variance that is proportional to the mean, where 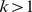 indicates degree of over dispersion. Similarly, *λ* equals two with 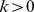 refers to a gamma model with the standard deviation that is proportional to the mean [22]. To explore a suitable value for *λ* to identify the right GLM model, we plotted center-specific log-transformed standard deviation versus center-specific log-transformed mean, separately for each of the three vegetable subgroups as reported on 24-HDR in the EPIC study. Then *λ* is estimated as twice the slope of the fitted regression line. The GLM model considered here can accommodate family of densities with skewed (asymmetric) distributions. We chose to use graphical method to identify *λ* due to its simplicity as opposed to estimation methods such as the maximum likelihood (MLE).

### Nonlinearity and variable transformation

The relation between dietary intake variables is often nonlinear. To explore the form of relation between consumption probability as reported on 24-HDR and usual intake as reported on DQ, we applied two techniques: the empirical logit plot, and the nonparametric generalized additive model (GAM). With the empirical logit technique, we categorized DQ measurements, starting with the category of never-consumers followed by 10 g/day intake intervals. In each category, we computed the logit of consumption as reported on 24-HDR. The formula for the empirical logit transformation [20], [23] of consumption used is given by
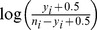
(5)where 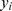 is the number of individuals who reported consumption on the 24-HDR and 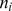 is the number of individuals in the i^th^ DQ-category. The addition of 0.5 to both the numerator and the denominator of the logit function serves to avoid indefinite empirical logit values when 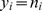 or 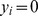, and this particular value minimizes the bias in estimating the log odds [20]. The estimated empirical logit is then plotted against the DQ category-specific means. We fitted a loess curve to the resulting scatterplots to have a visual inspection of the form of relation between the two variables [24]. We further made the empirical logit plots for each of the participating country in the EPIC study. With the nonparametric GAM technique, we obtained an optimal smoothing splines for the relation between the consumption probability, as reported on 24-HDR, and DQ and other continuous variables based on generalized cross validation criterion (GCV) [25]. We fitted the GAM model for consumption probability, assuming a binomial response and a logit link function using the mcgv package in R [26]. In the GAM model, we included confounding variables in the disease model (**Z**). We used the partial prediction plot from the smoothed DQ component to identify plausible forms of parametric transformations for the DQ [27]. From the selected set of parametric transformations, Akaike Information Criterion (AIC) was used to identify the optimal transformation. Similar to the consumption probability part, we explored optimal form of DQ for the consumed amount part of the calibration model with the GAM approach.

### Variables inclusion in the calibration model

The theory of regression calibration states that all confounding variables in the disease model must also be included in the calibration model in addition to the covariates that only predict dietary intake [14]. We used the same set of confounding variables in Agudo [3] that studied the relation between intake of vegetables and mortality in the Spanish cohort of EPIC. The eight confounding variables were: BMI (kg/m^2^), smoking status (never, former, current smoker), physical activity index (inactive, moderately inactive, moderately active, active), lifetime alcohol consumption (g/day), level of education (none, primary, technical, secondary, university), age at recruitment (years), total energy (kcal), and sex (male/female).

The covariates that only predict intake as measured 24-HDR were selected based on their statistical significance in the calibration model (3). We included plausible two-way interaction terms of DQ measurements with the other covariates in the calibration model. We hereafter refer to each of the calibration model with covariates selected using the standard theory with the prefix “standard”, here, standard two-part calibration model. The covariates are not only included once but twice in the two-part calibration model (i.e., in each part of the two-part model), thus posing a threat to over fitting. Moreover, some disease confounding variables might not necessarily predict true usual intake conditional on DQ. We therefore conducted a backward elimination on the standard two-part calibration model based on a significance level α of 0.2. We chose 0.2 to ensure that no significant covariates are excluded from the model. We hereafter refer to each of the reduced version of the standard calibration model with the prefix “reduced”, here, reduced two-part calibration model.

To assess the power of the probability part of the two-part calibration model to correctly discriminate consumers from non-consumers as reported by 24-HDR, we used the Area under the curve from the Receiver operating characteristic curve of the fitted logistic model [28]. For the consumed amount part, we assessed the predictive power of the model based on the root mean squared error and the mean bias [29]. In building the two-part calibration model, we conducted country-specific rather than center-specific regression calibration models to obtain stable estimates given the relatively smaller sample sizes in each center [10].

We also fitted other forms of regression calibration models to compare with the developed two-part calibration model. These forms of the calibration model include:

- A two-part calibration model similar to the developed one but with untransformed DQ. We hereafter refer to this model as “Two-part (untransformed DQ)”. The aim of fitting this model was to assess the effect of nonlinearity on the performance of a two-part calibration model.
- A one-part calibration model with untransformed DQ and with the usual assumptions of a classical linear model. This is the calibration model commonly used by epidemiologists to adjust for the bias in the diet-disease association. In this model, two strong assumptions are made, namely, normality and linearity. The aim of fitting this calibration model was to quantify the inadequacy in adjusting for the bias in the diet-disease association when these assumptions are violated.

In each of the two forms of calibration models, we used the same set of covariates in each part of the standard two-part calibration but with different parametric forms of DQ as explained above. We conducted a backward elimination (α = 0.2) on each of these forms of regression calibration models to obtain their reduced forms. Subsequently, we used a Cox proportional hazard model to study the association between usual intake of vegetable subgroups and all-cause mortality [30]. The Cox proportional hazards model was stratified by center and sex. To explore the form of relation between usual intake of each of the three vegetable subgroups and all-cause mortality in the Cox model, we plotted the log hazard ratio estimate against the DQ category-specific median intake [31].

We used bootstrap procedure to compute correct standard error for the log hazard ratio estimate. The bootstrap approach accounts for the uncertainty in the calibration process. We used center-stratified bootstrap procedure on the calibration sub-study. To each bootstrap sample, we added the main-study data and fitted regression calibration model to generate replicate versions of 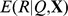 for each subject in the entire EPIC cohort [32]. To each replicate data, we fitted the Cox model yielding an estimate of log hazard ratio with a standard error. The within-calibration and between-calibration variances were combined using Rubin’s formula to account for the uncertainty in the calibration process [33]–[35]. The Rubin’s formula used to estimate the standard error for the log hazard ratio estimate is
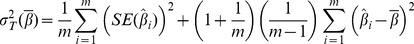
(6)where 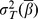 is the total variance of the mean of log hazard ratio estimate from m calibrated samples, 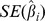 is the within-calibration standard error, and 
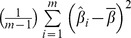
 is the between-calibration variance.

We fitted a Cox proportional hazards model that ignores the measurement error in DQ. This method is hereafter referred to as the naïve method. In the naïve method, the DQ measurements were used to study the association between usual intake of a vegetable subgroup and all-cause mortality.

## Results

### Excess zeroes, heteroscedasticity and skewness in reference measurements

**Figure 1:**
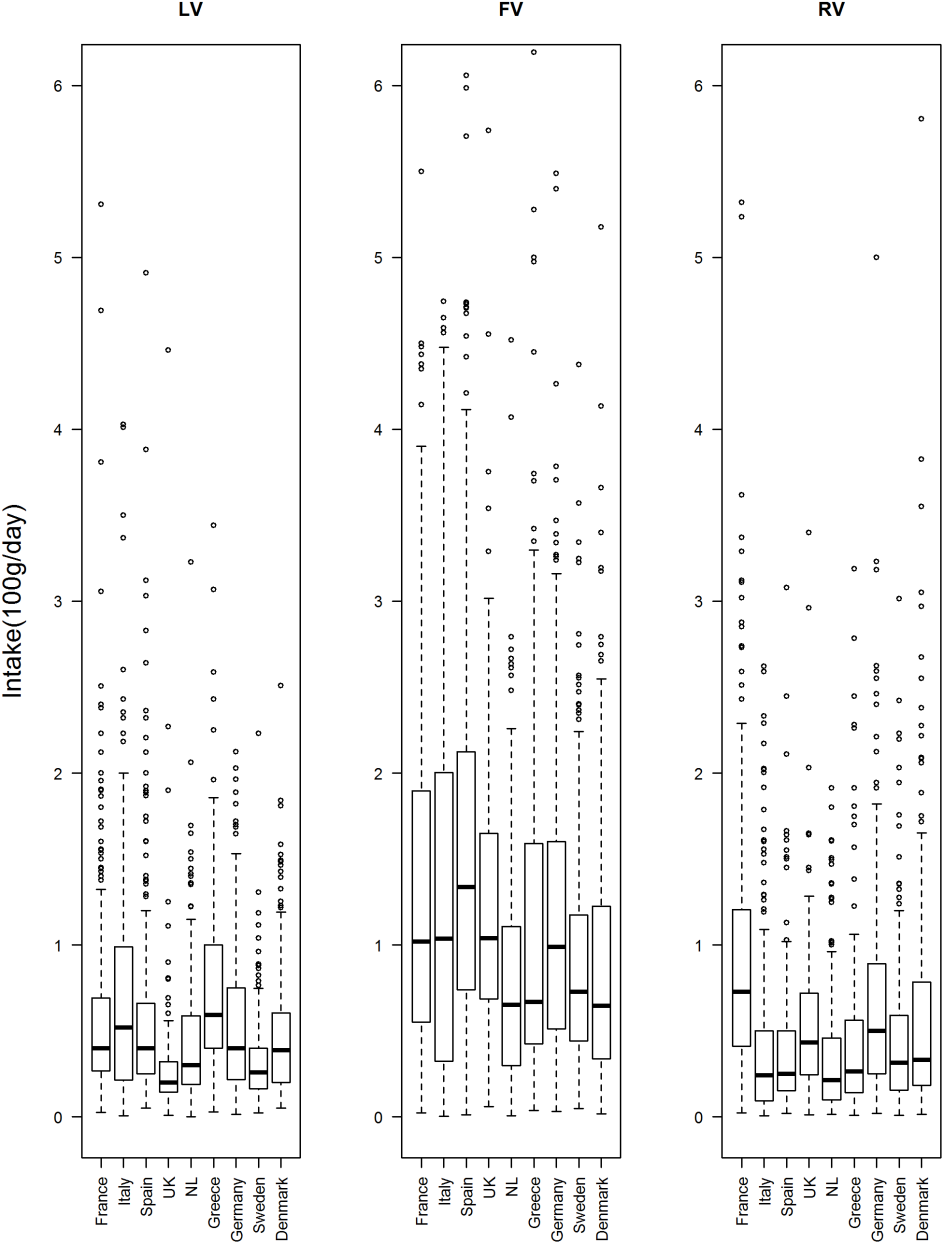
The boxplots for the distribution of intake of vegetable subgroups. The country-specific boxplots show the distribution of the consumed amount for those who reported consumption on the 24-HDR for leafy vegetables (LV), fruiting vegetables (FV) and root vegetable (RV) subgroups in the EPIC study, 1992–2000.

In Table 1, each of the three vegetable subgroups showed a high percentage of zero reference measurements as reported on the 24-HDR, especially for root vegetable subgroup in most of the participating countries. The rather high percentage of zero reference measurements suggests that these subgroups of vegetables are not consumed daily by everyone. The Pearson correlation coefficient for each of the three vegetable subgroups in each of the participating countries, as measured with 24-HDR and DQ, were rather low but mostly statistically significant. The boxplots for the distribution of the consumed amount on consumption events as reported on 24-HDR showed positive skewed distributions for these dietary variables (Figure 1). These exploratory findings suggested a need to properly handle the excess zeroes, to choose either a suitable distribution or a correct transformation for the consumed amount, as reported on 24-HDR in building a calibration model.

**Table 1:** Country-specific summary measures for the percentage of zero intake measurements reported on 24-HDR (% R = 0, non-consumers) and Pearson Correlation (ρ) for intake as measured by 24-HDR and DQ for leafy vegetables, fruiting vegetables and root vegetables.

	Leafy vegetables			Fruiting vegetables		Root vegetables	
Participating Countries	N	% R = 0	ρ	% R = 0	ρ	% R = 0	ρ
France	4735	42.8	0.17	44.4	0.10	71.6	0.06
Italy	3961	59.3	0.16	37.6	0.15	79.6	0.11
Spain	3220	48.9	0.34	31.7	0.22	76.1	0.12
UK	1313	68.2	0.16	40.8	0.19	59.3	0.23
Netherlands	4545	70.5	0.10	48.7	0.21	82.0	0.14
Greece	2930	67.9	0.10	29.5	0.13	83.2	0.03^ns^
Germany	4418	75.9	0.15	41.6	0.17	79.2	0.22
Sweden	a6132	70.5	0.19	34.9	0.24	67.2	0.17
Denmark	3918	77.4	0.09	41	0.21	61.8	0.40
Norway	b1798					58.5	0.12

EPIC Study, 1999–2000.

a N is 3132 instead of 6132 for leafy vegetables in Sweden because data from Umeå were excluded from analysis based of the inclusion criteria in EPIC;

b N refers to data for root vegetables only because data for Norway were excluded for leafy vegetable and fruit vegetable subgroups; ^ns^means correlation is not statistically significant at α = 0.05, other correlation coefficients are highly significant with P<0.0001.

**Figure 2:**
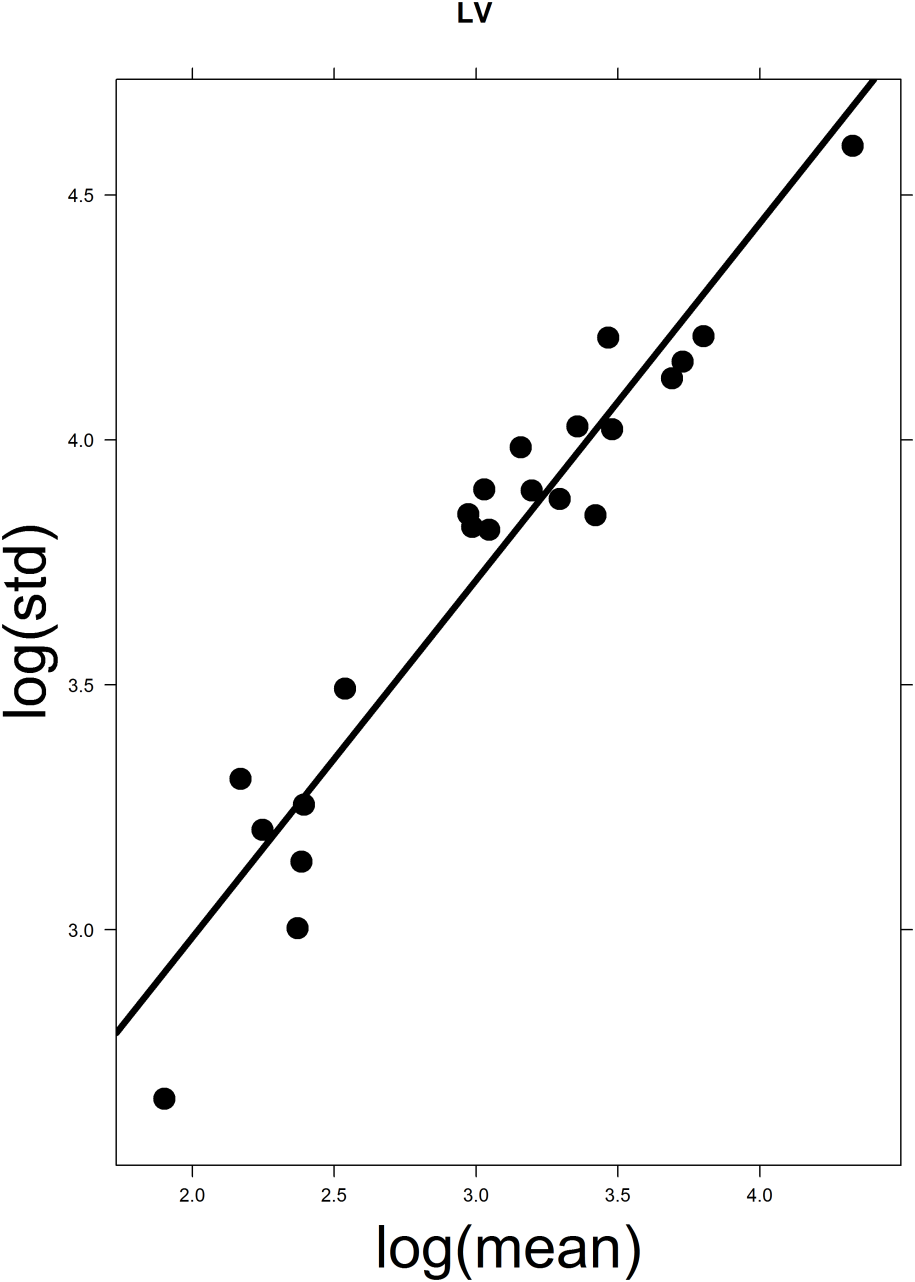
The variance-mean relation for Leafy vegetable intake. The graph shows a least squares regression line fitted to the scatterplots of the logarithm of center-specific standard deviation versus logarithm of center-specific mean of the consumed amount of leafy vegetables for those who reported consumption on the 24HDR in the EPIC Study, 1992–2000. The approximately linear regression line suggests a variance that increases with the mean.

For each of the three vegetable subgroups, a linear trend was shown between the log of standard deviation and the log of the mean for the consumed amount (see Figure 2 for leafy vegetables). The linear trend is a clear evidence of a variance that increases with a mean (presence of heteroscedasticity). The slope (standard error) of least squares regression line fitted to the resulting scatterplots was estimated as 1.057 (0.085). For fruiting vegetables, the estimates were 0.994 (0.076) as shown in Figure S1. Likewise for root vegetables, the estimates were 1.021 (0.130) as shown in Figure S2. These slopes of the fitted lines were all close to the theoretical value of 1 for a GLM gamma model. Based on these exploratory findings, we chose a gamma GLM model for the consumed amount part of the two-part calibration model separately for each of the three vegetable subgroups. The correlation between each of the three vegetable subgroups ranged from 0.06 to 0.12 with total energy and from −0.07 to 0.05 with alcohol, as measured with DQ. These low correlations suggest minimal contamination effect of measurement error, hence justifying our choice not to adjust for the error in these variables.

### Non-linearity and variable transformations

**Figure 3:**
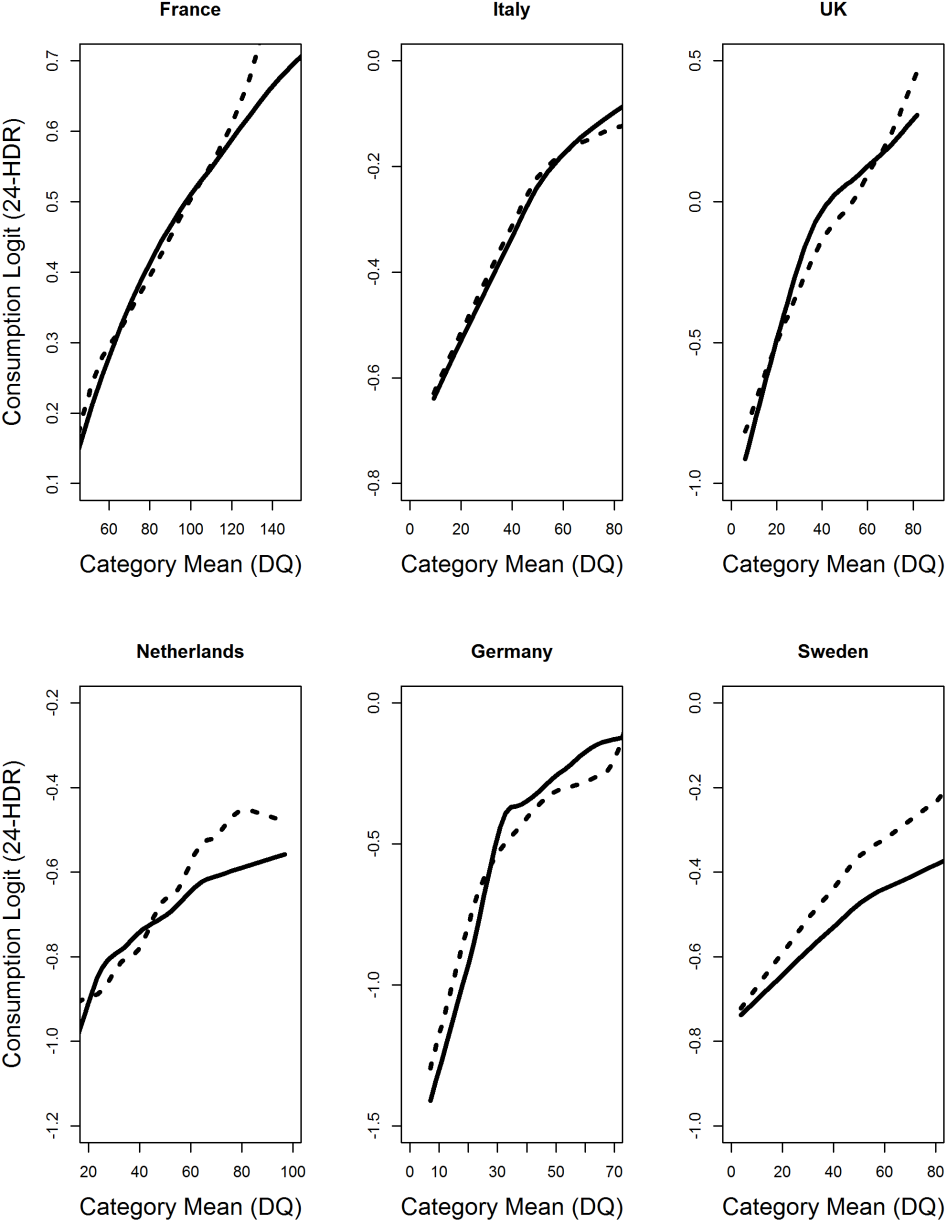
The empirical logit graph for Leafy vegetable intake. The graph shows loess curves fitted to 1) the scatterplots for the empirical logit (dotted line) and 2) the mean of the predicted logit from a logistic model with log-transformed DQ (thick line) against the DQ category-specific means for leafy vegetable intake in the EPIC Study, 1992–2000. The similarity in the two logit curves suggests that a log- transformed DQ is appropriate for the consumption probability part of the two-part calibration model.

To explore the form of DQ in the part I model for the consumption probability, the loess curve fitted to the scatterplots of the empirical logit versus the DQ category-specific means presented in Figure 3 showed a nonlinear relation between the logit of consumption as reported on 24-HDR and the DQ reported intake (dotted lines). The partial prediction plots from the GAM approach showed similar behavior. From the plausible set of parametric transformations for DQ, here, square-root and logarithmic, we chose log-transformed DQ based on the AIC criterion for each model fitted to country-specific data. As a result, we further fitted a logistic model with log-transformed DQ and computed mean of the predicted logit of consumption in each category of DQ. The loess curve fitted to the scatterplots of the mean of predicted logit against DQ category-specific means is shown in the same figure (continuous line). The similarity of the two loess curves suggested the aptness of log-transforming DQ in the part I model for consumption probability of leafy vegetables. The graphs for fruiting vegetables and root vegetables yielded similar results.

To explore the form of DQ in the part II model for the consumed amount part, we fitted a GAM model with gamma distributed error terms and a log link function (as suggested by exploratory results). Based on partial prediction plots for the smoothed DQ components and using the AIC criterion, we chose a square-root transformed DQ for both leafy vegetables and root vegetables subgroups, and a log-transformed DQ for fruiting vegetables.

### Two-part regression model building

In addition to the confounding variables in the Cox model (under variables inclusion in the calibration model sub-section), season of DQ administration, center where the DQ was administered and the body weight of the participant were also included in the calibration model because they predicted intake of each of the three vegetable subgroups. Other covariates included in the standard two-part calibration model were the transformed DQ, and two-way interaction of transformed DQ with sex, age, season, BMI and center. We used the same set of covariates on each part of the standard two-part calibration model but with additional quadratic term for age at recruitment in the consumed amount part. In Table 2, we showed the remaining significant terms after a backward elimination on each part of the standard two-part calibration model separately for each of the three vegetable subgroups.

**Table 2:** Significant covariates (marked ×) in the reduced two-part calibration models, after a backward elimination on each part of the standard two-part regression calibration model with transformed DQ and with other covariates selected using the standard way of variable inclusion.

	Leafy vegetables		Fruitingvegetables		Root vegetable	
Covariates	Part I	Part II	Part I	Part II	Part I	Part II
**Main effects**						
Q^t^	×	×	×	×	×	×
BMI		×	×	×	×	×
Smoking status	×		×	×	×	×
Physical activity		×	×	×		×
Lifetime alcohol	×		×			
Education	×	×	×	×	×	
Age	×	×	×	×	×	×
Age^2^				×		
Total energy			×	×		×
Weight			×	×		×
Center	×	×	×	×	×	×
Season		×	×	×	×	×
Sex	×	×		×	×	
**Interaction** **terms**						
Q^t^ * sex		×			×	
Q^t^ * age	×	×		×		×
Q^t^ * season			×		×	
Q^t^ * BMI				×	×	
Q^t^ * center	×	×	×	×	×	×

EPIC Study, 1992–2000.

Q^t^ is a transformed DQ; Part I, refers to consumption probability part of the two-part calibration model; Part II, refers to consumed amount part of the two-part calibration model;

*refers to an interaction term.

The areas under the curve from the ROC curve for the consumption probability part of the standard two-part calibration model and its reduced form were quite similar for each of the vegetable subgroups (Table 3). This suggest that some confounding variables and other two-way interaction terms of DQ with other covariates in the standard model do not necessarily predict the consumption probability and therefore should not be included in the calibration model.

**Table 3:** The area under the curve (AUC) from ROC curve for consumption probability (Part I), and root mean square error (RMSE) and mean bias for the consumed amount (Part II) of the standard and the reduced forms of two-part regression calibration models with transformed DQ.

Vegetable Subgroups	Part I		Part II	
	Models	AUC	RMSE^a^	Mean Bias^b^
Leafy	Standard	0.6846	66.841	0.0223
	Reduced	0.6843	64.578	0.0019
Fruiting	Standard	0.6305	118.823	0.0446
	Reduced	0.6304	110.415	−0.0334
Root	Standard	0.6413	68.626	0.0895
	Reduced	0.6408	66.524	0.0883

a
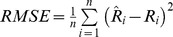
; ^b^ 
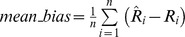

A similar remark could be made for the consumed amount part of the model, based on the root mean squared error and the mean bias, which were quite similar.

**Figure 4:**
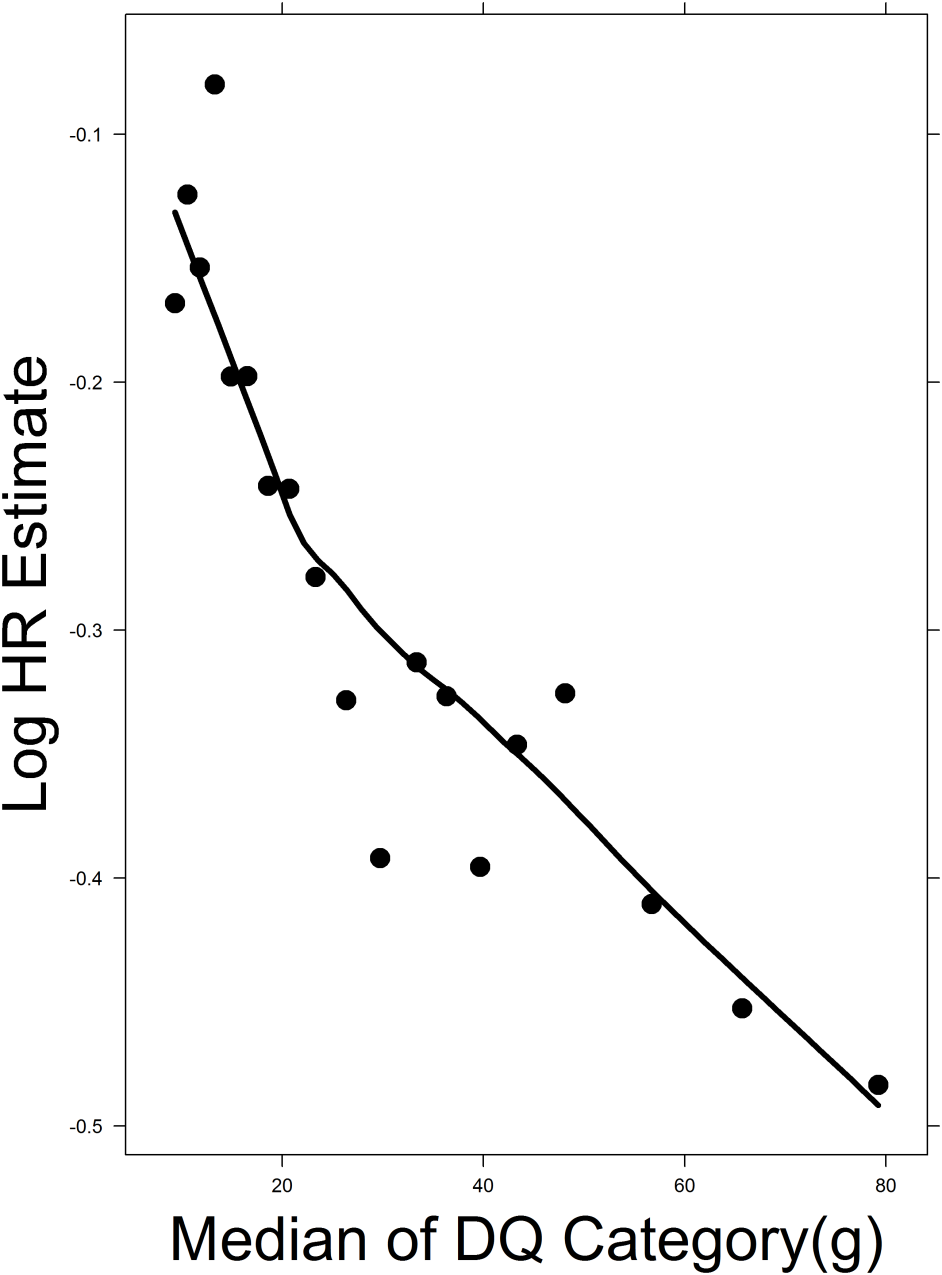
Linearity assessment in the Cox proportional hazards model for Leafy vegetables. The graph shows a smoothed curve fitted to the scatterplots of log hazard ratio estimate of leafy vegetable intake on all-cause mortality in each DQ category versus DQ category-specific median intake. The approximately linear downward trend suggests a possible linear relation and a beneficial effect of vegetable intake on the risk of all-cause mortality.

The graphical exploration of the smoothed curve fitted to the scatterplots of the log hazard ratio estimate of dietary intake on all-cause mortality versus the DQ category-specific median intake showed approximately linear relations for each of the three vegetable subgroups as shown in Figure 4 for leafy vegetables, Figure S3 for fruiting vegetables, and Figure S4 for Root vegetables. We therefore assumed a linear term for DQ in the three fitted Cox proportional hazards models.

As expected, the log hazard ratio estimate for usual intake in the Cox model adjusted for measurement error in DQ were larger in absolute value than the naive estimate that ignores the measurement error. Similar remark was made for all the fitted forms of regression calibration models but the standard two-part calibration model with untransformed DQ (Table 4).The log hazard ratio estimates adjusted for the bias with the standard calibration models were smaller than those adjusted with the reduced calibration models, e.g., −0.265 for the standard two-part (transformed DQ) and −0.479 for the reduced two-part (transformed DQ) calibration model per 100 g intake of root vegetables. The poor performance of the standard calibration models might be due to over fitting by covariates that did not significantly predict usual intake of vegetable sub-groups. The log hazard ratio estimate adjusted with the standard two-part calibration model was even smaller than the naïve estimate. This shows that a poorly specified calibration model can result in adjusted association estimates that are more biased than the unadjusted estimates. The standard error of the log hazard ratio estimate corrected for the uncertainty in the calibration was larger than the uncorrected one for each of the calibration models presented. This means that ignoring uncertainty in the calibration underestimates the standard error. The underestimation of standard error was more severe for the standard calibration models. Further, the log hazard ratio estimate calibrated with the reduced one-part linear calibration model was smaller than that obtained with the reduced two-part (transformed DQ) model. The seemingly poor performance of one-part linear calibration model suggests that a poorly specified calibration model does not adequately adjust for the bias in the diet-disease association. Further, the predicted intake values for some subjects not in the calibration sub-study, in some cases were rather unrealistic. The unrealistic predictions were mainly from the standard calibration model with untransformed DQ. The calibration models with untransformed DQ resulted in a much smaller log hazard ratio estimate than their counterparts with transformed DQ. This might be driven by extreme prediction from highly skewed DQ measurements in the calibration model. The effect of the extreme DQ values was further compounded by two factors: including the same covariate twice in the two-part calibration model and by the exponentiation effect due to the log link function used to fit the calibration model. As a result, we conducted a small sensitivity analysis where the unreasonably high predicted values were retained in the Cox model. Including these high predicted values resulted in massive change in the log hazard ratio estimate mainly with standard two-part calibration model with untransformed DQ. For leafy vegetables, for instance, including the unrealistic predictions from the standard two-part calibration model with untransformed DQ changed the estimate of log hazard ratio from −0.174 to −0.00518 per 100 g intake. In Table S1, we present the percentages of these unrealistic predictions, defined as extreme if it exceeded fivefold the ninety ninth percentile of the predicted usual intake. In the final analysis, we excluded these unrealistic values.

**Table 4:** Log hazard ratio estimate (standard error) per 100 g usual intake of each of the three vegetable subgroups, calibrated with each of the three forms of regression calibration models in their reduced and standard forms.

		Reduced form				Standard form				
VegetableSubgroups	Calibration methods	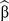 (s.e^a^; s.e^b^)		s.e ratio^c^		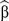 (s.e^a^; s.e^b^)		s.eratio^c^		
Leafy	Naïve method		−0.144 (0.027)				−0.144 (0.027)			
	One-part linear calibration		−0.480 (0.090;0.112)		1.24		−0.409 (0.083;0.127)		1.53	
	Two-part (untransformedDQ)		−0.395 (0.092;0.183)		1.99		−0.174 (0.089;0.278)		3.11	
	Two-part (transformedDQ)		−0.509 (0.090;0.292)		3.24		−0.461 (0.047;0.160)		3.41	
Fruiting	Naïve method		−0.094 (0.014)				−0.094 (0.014)			
	One-part linear calibration		−0.125 (0.031;0.034)		1.11		−0.123 (0.031;0.034)		1.11	
	Two-part(untransformedDQ)		−0.161 (0.030;0.034)		1.14		−0.109 (0.030;0.073)		2.42	
	Two-part (transformedDQ)		−0.255 (0.037;0.108)		2.92		−0.228 (0.035;0.131)		3.74	
Root	Naïve method		−0.160 (0.026)				−0.16 (0.026)			
	One-part linear calibration		−0.342 (0.060;0.082)		1.36		−0.305 (0.054;0.077)		1.43	
	Two-part(untransformedDQ)		−0.203 (0.088;0.219)		2.49		−0.107 (0.060;0.167)		2.78	
	Two-part (transformedDQ)		−0.479 (0.070;0.214)		3.06		−0.265 (0.056;0.181)		3.23	

s.e^a^ is the standard error (×10^−2^) for 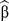 that does not account for the uncertainty in the calibration; s.e^b^ is the standard error (×10^−2^) that accounts for the uncertainty in the calibration; s.e ratio^c^ is the ratio of s.e^b^ to s.e^a^.

## Discussion

In this work, we adapted a two-part regression calibration model initially developed for multiple 24-HDR measurements per individual for episodically consumed foods to a single replicate setting. We focused on dietary intake data that are skewed, heteroscedastic, and with substantial percentage of zeroes as reported on the 24-HDR. We further described how to explore and identify a suitable GLM model and a correct parametric form of a continuous covariate in the calibration model. As a result, we applied flexible GLM models that could simultaneously handle the skewness and heteroscedasticity in the consumed amount. Thus, we avoided complications resulting from data transformation. We chose the log link function to stabilize the variance and to ensure positive prediction for usual intake [36].

The standard way of including variables in the calibration model states that all confounding variables in the disease model and those that only predict dietary intake but not the disease occurrence must be included in the calibration model. Given the complexity of the two-part calibration model, some confounding variables in the disease model do not necessarily predict dietary intake. This could pose a threat to over fitting the calibration model. We further conducted a backward elimination on each part of the two-part calibration model separately. The reduced calibration model with only significant covariates outperformed its standard counterpart in adjusting for the association bias. Leaving out confounding variables from the calibration model is against the standard theory of regression calibration. Nevertheless, we argue that if the omitted covariates have no effect in the calibration model, they should be excluded and the calibration method should still be correct. We further found out that assuming linearity when it does not hold in a calibration model can pose a serious threat to the bias-adjustment of the association parameter. The association parameter estimate adjusted for the bias with a poorly specified calibration model can sometime be worse than the unadjusted estimates. Thoresen [37] also found, in a simulation study, that a less accurately specified calibration model can have a considerable impact on the degree of bias-adjustment. We observed that predicted values for some subjects not in the calibration sub-study were extremely large. The extreme predictions resulted mainly from standard calibration models with linear DQ as a covariate. In such a case, predictions are made outside the variable space on which the model is fitted. Due to the curse of dimensionality, the prediction space would extend more outside the variable space in the complex models.

The consumption probability and the consumed amount for episodically consumed foods may be correlated. In each of the fitted two-part calibration models, we accounted for this correlation partly by allowing covariates to overlap on both parts of the calibration model [14]. With only a single 24-hour recall measurement per subject, any further correlation cannot be estimated. In future studies, a sensitivity analysis can be performed to assess the effect of the unaccounted part of the correlation. This can be done by varying the magnitude of the assumed positive correlation between the consumption probability and the consumed amount.

A limitation of this study is that we made some strong assumptions. First, we assumed the 24-HDR to be unbiased measurement of true usual intake. Second, we assumed that the errors in the 24-HDR are uncorrelated with the errors in the DQ. However, previous studies have shown that these assumptions may not hold for dietary self-report instruments, and that, use of 24-HDR as a reference instrument for vegetable intake may be flawed [4], [38]–[40]. The biomarker studies using doubly labelled water for energy intake and urinary nitrogen for protein intake suggest that self-reports on recalls or food records may be biased. This is because individuals may systematically differ in their reporting accuracy. Additionally, the errors in these short-term instruments are shown to be positively correlated with the errors in the DQ [41]. As a result, using 24-HDR as a reference instrument can seriously underestimate true attenuation [42]. Therefore, the results obtained with the 24-HDR as reference instrument should be interpreted with caution. Nevertheless, the bias in 24-HDR is reported to be substantially less severe than that in the DQ [38]. Thus, when there is no objective biomarker measurements for dietary intake, using 24-HDR may still provide the best possible estimation of true intake [14].

In summary, a correctly specified two-part regression calibration model, which fits the data better, can adequately adjust for the bias in the diet-disease association, when only a single reference measurement is available per individual. Further, the ability to adjust for the bias is influenced considerably by the form of the specified calibration model. We therefore advise researchers to pay special attention to calibration model specification, with respect to the response distribution and the form of the covariates.

## Supporting Information

Figure S1 **The variance-mean relation for Fruiting vegetables (FV).** The graph shows a least squares regression line fitted to the scatterplots of the logarithm of center-specific standard deviation versus logarithm of center-specific mean of the consumed amount of fruiting vegetables for those who reported consumption on the 24HDR in the EPIC Study, 1992–2000. The approximately linear regression line suggests a variance that increases with the mean.
(TIFF)

Figure S2 **The variance-mean relation for Root vegetables (RV).** The graph shows a least squares regression line fitted to the scatterplots of the logarithm of center-specific standard deviation versus logarithm of center-specific mean of the consumed amount of root vegetables for those who reported consumption on the 24HDR in the EPIC Study, 1992–2000. The approximately linear regression line suggests a variance that increases with the mean.
(TIFF)

Figure S3 **Linearity assessment in the Cox proportional hazards model for Fruiting vegetables.** The graph shows a smoothed curve fitted to the scatterplots of log hazard ratio estimate of fruiting vegetable intake on all-cause mortality in each DQ category versus DQ category-specific median intake. The approximately linear downward trend suggests a possible linear relation and a beneficial effect of fruiting vegetable intake on the risk of all-cause mortality.
(TIF)

Figure S4 **Linearity assessment in the Cox proportional hazards model for Root vegetables.** The graph shows a smoothed curve fitted to the scatterplots of log hazard ratio estimate of root vegetable intake on all-cause mortality in each DQ category versus DQ category-specific median intake. The approximately linear downward trend suggests a possible linear relation and a beneficial effect of root vegetable intake on the risk of all-cause mortality.
(TIF)

Table S1 **Unrealistic predicted usual intake of vegetable subgroups.** The table displays the maximum and the ninety-ninth percentile of predicted usual intake and percentage (number) of unrealistic predictions (i.e., unrealistic if greater than five times ninety-ninth percentile of predicted intake) using different forms of regression calibration models; each model in its standard form, that is, with the covariates selected using the standard theory, and also in the reduced form, that is, with covariates that significantly predict intake.
(DOC)
