# Supplementary figures and images for: Use of Two-Part Regression Calibration Model to Correct for Measurement Error in Episodically Consumed Foods in a Single-Replicate Study Design: EPIC Case Study
Source: PLoS One. 2014 Nov 17;9(11):e113160. doi: 10.1371/journal.pone.0113160 (PMC4234679; doi:10.1371/journal.pone.0113160)

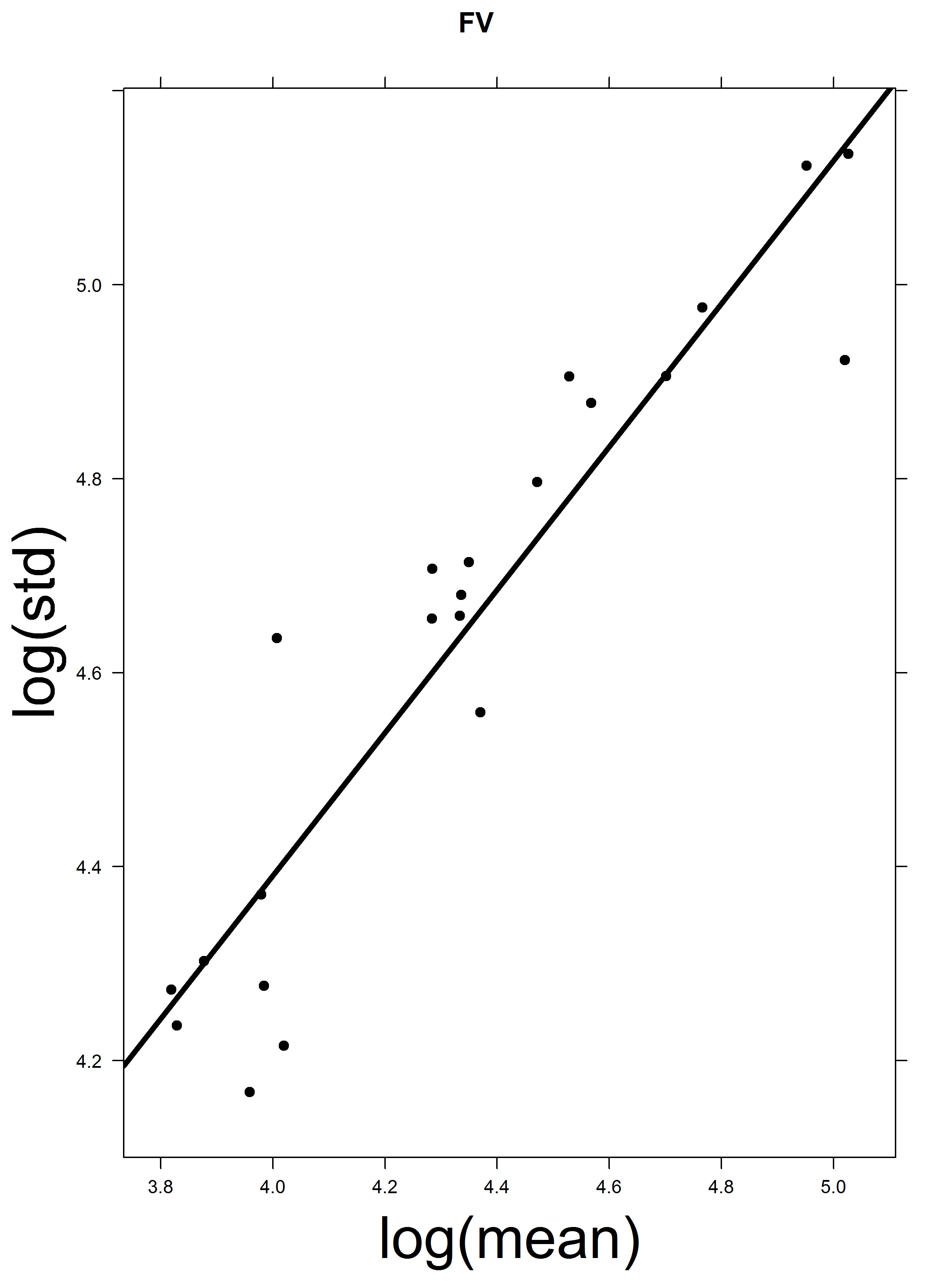

Supplement: Figure S1 — The variance-mean relation for Fruiting vegetables (FV). The graph shows a least squares regression line fitted to the scatterplots of the logarithm of center-specific standard deviation versus logarithm of center-specific mean of the consumed amount of fruiting vegetables for those who reported consumption on the 24HDR in the EPIC Study, 1992–2000. The approximately linear regression line suggests a variance that increases with the mean. (TIFF) [file pone.0113160.s001.tiff]

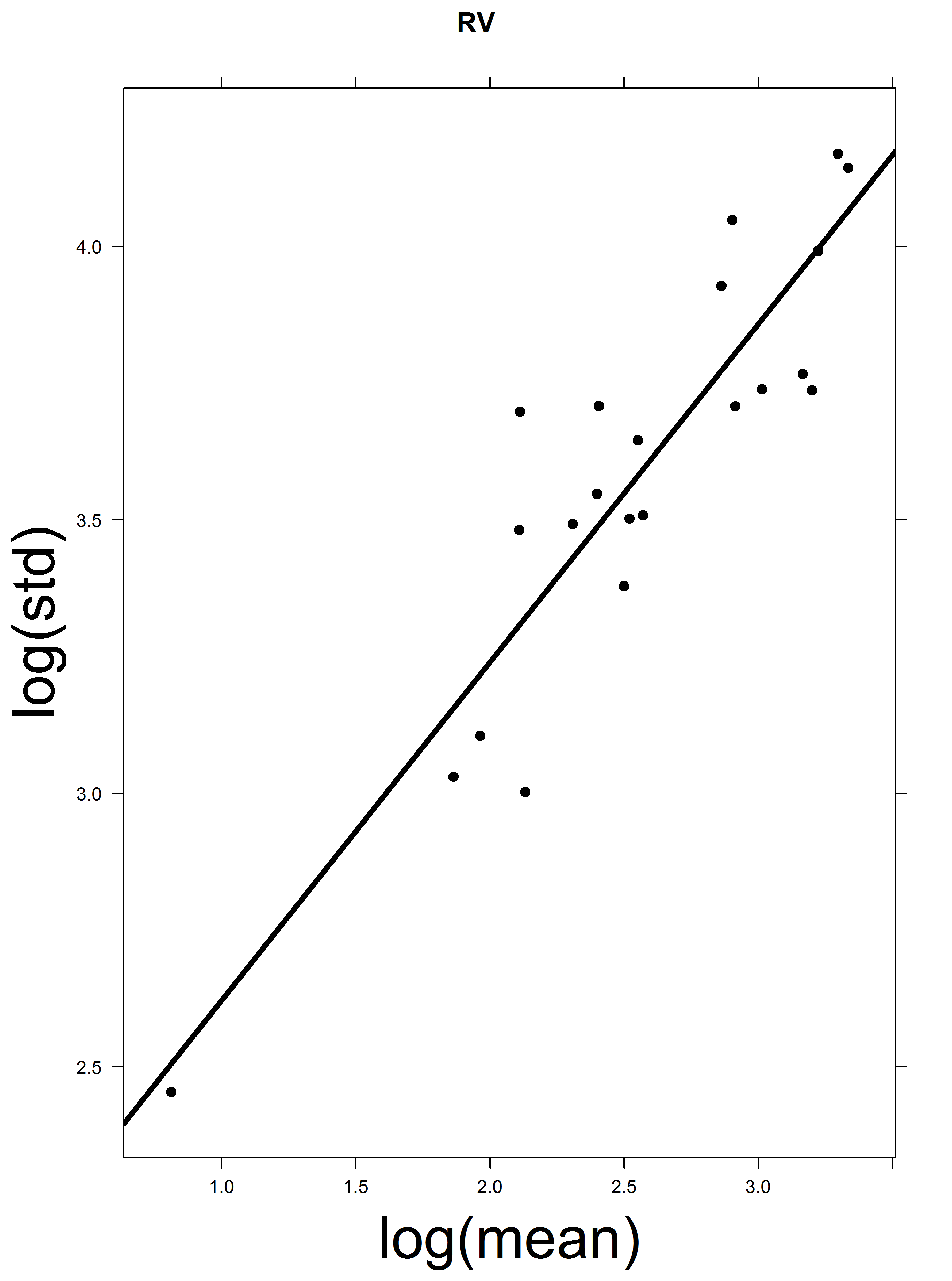

Supplement: Figure S2 — The variance-mean relation for Root vegetables (RV). The graph shows a least squares regression line fitted to the scatterplots of the logarithm of center-specific standard deviation versus logarithm of center-specific mean of the consumed amount of root vegetables for those who reported consumption on the 24HDR in the EPIC Study, 1992–2000. The approximately linear regression line suggests a variance that increases with the mean. (TIFF) [file pone.0113160.s002.tiff]
