# Supplementary material for: Use of Two-Part Regression Calibration Model to Correct for Measurement Error in Episodically Consumed Foods in a Single-Replicate Study Design: EPIC Case Study
Source: PLoS One. 2014 Nov 17;9(11):e113160. doi: 10.1371/journal.pone.0113160 (PMC4234679; doi:10.1371/journal.pone.0113160)
Supplement: Table S1 — Unrealistic predicted usual intake of vegetable subgroups. The table displays the maximum and the ninety-ninth percentile of predicted usual intake and percentage (number) of unrealistic predictions (i.e., unrealistic if greater than five times ninety-ninth percentile of predicted intake) using different forms of regression calibration models; each model in its standard form, that is, with the covariates selected using the standard theory, and also in the reduced form, that is, with covariates that significantly predict intake. (DOC) [file pone.0113160.s005.doc]

Table S1. Unrealistic predicted usual intake of vegetable subgroups. The maximum, the ninety-ninth percentile of predicted usual intake, and percentage (number) of unrealistic predictions using different forms of regression calibration models, each model in its reduced and standard forms

| Vegetable  subgroup | Calibration method | | Reduced form | | | | Standard form | | | |
| --- | --- | --- | --- | --- | --- | --- | --- | --- | --- | --- |
|  |  | Max | | P99 | %(n) |  | | Max | P99 | %(n) |
| Leafy | One-part linear calibration | 224 | | 76.77 | 0.00(0) |  | | 353 | 78.79 | 0.00(0) |
|  | Two-part (untransformed DQ) | 36292 | | 133.7 | 0.00(12) |  | | 1.2×10^10^ | 86.08 | 0.04(171) |
|  | Two-part (transformed DQ) | 987.35 | | 132.8 | 0.00(1) |  | | 13162.8 | 138.16 | 0.00(20) |
| Fruiting | One-part linear calibration | 618 | | 197 | 0.00(0) |  | | 591 | 200.66 | 0.00(0) |
|  | Two-part (untransformed DQ) | 3401.66 | | 254.3 | 0.01(50) |  | | 3879 | 253.6 | 0.02(85) |
|  | Two-part (transformed DQ) | 487.10 | | 240 | 0.00(0) |  | | 511 | 206.96 | 0.00(0) |
| Root | One-part linear calibration | 540.40^a^ | | 65 | 0.00(0) |  | | 552.202 | 70.639 | 0.00(0) |
|  | Two-part(untransformed DQ) | 1.25×10^10^ | | 124.9 | 0.02(82) |  | | 2.3×10^10^ | 132.23 | 0.02(89) |
|  | Two-part (transformed DQ) | 2384.99 | | 126.5 | 0.00(1) |  | | 27419.9 | 130.4 | 0.00(3) |

Standard form: covariates selected based on standard theory, that is, with all disease confounding variables and covariates that only predict intake but not risk of the disease.

Reduced form: obtained by a backward elimination on the standard calibration model with α=0.2.

Max: maximum predicted value; p99 is ninety ninth percentile of predicted intake.

^a^ the maximum value is not considered unrealistic despite being greater than fivefold ninety ninth percentile of predicted usual intake because it is within WHO recommendation of 400g daily intake.
